# Effects of Angiotensin-Converting Enzyme Inhibitors on Arterial Stiffness: A Systematic Review and Meta-Analysis of Randomized Controlled Trials

**DOI:** 10.1155/2020/7056184

**Published:** 2020-02-25

**Authors:** Xiuli Li, Peng Chang, Qiongying Wang, Hao Hu, Feng Bai, Ningyin Li, Jing Yu

**Affiliations:** Department of Cardiology, Lanzhou University Second Hospital, Lanzhou, China

## Abstract

To determine the effects of ACEIs on arterial stiffness, a meta-analysis of randomized controlled trials was conducted. Relevant articles that investigated the effects of ACEIs on arterial stiffness from PubMed, Embase, and the Cochrane library from inception to September 2018 were systematically retrieved. The investigated outcomes included brachial-ankle pulse wave velocity (ba-PWV) and carotid-femoral PWV (cf-PWV) by using weighted mean differences (WMDs) and 95% confidence intervals (CIs) with the random-effects model. A total of 17 RCTs including 1,458 individuals were included. The summary results indicated no significant differences between ACEIs and control for ba-PWV and cf-PWV. Also, no significant differences between ACEI and control for ba-PWV and cf-PWV were observed in hypertensive patients, while the therapeutic effects of ACEI versus placebo showed statistically significant difference. Moreover, subgroup analysis indicated that the levels of ba-PWV were significantly associated if the study was conducted in Western countries, mean age <60.0 years, percentage male ≥60.0%, compared with ARBs, baseline PWV <10.0, and high-quality study. Furthermore, the significant levels of cf-PWV in patients who received ACEIs were observed when percentage male was ≥60.0% and the studies were of high-quality. Finally, no significant differences were observed between ACEIs and other antihypertensive drugs regarding the changes of systolic blood pressure (SBP) and diastolic blood pressure (DBP). The overall analysis suggested no significant differences between ACEIs and other antihypertensive drugs for ba-PWV and cf-PWV levels, whereas ACEIs versus placebo showed lower levels of ba-PWV and cf-PWV.

## 1. Introduction

Cardiovascular disease is one of the major diseases that seriously threaten the health of several people worldwide [1]. Currently, there are several studies that have illustrated the pathological changes of the vascular wall that underlie cardiovascular events and play an important role in the progression of cardiovascular diseases [2–4]. Furthermore, changes in the structure of arterial wall caused declination in arterial compliance that might precede the clinical symptoms of the disease [5–7]. Therefore, arterial elasticity and function are used as a variety of cardiovascular risk factors for subclinical vascular lesions [8–10]. Recently, it has been demonstrated that arterial stiffness is another important risk factor that is independent of other cardiovascular risk factors and is considered to be an alternative endpoint of cardiovascular disease, and is associated with the morbidity and mortality of cardiovascular disease [11–13].

According to a previous study, angiotensin II is a potent vasoactive peptide in endothelial renin-angiotensin system (RAS), and angiotensin receptor blockers lower blood pressure (BP) and improve arterial elasticity [14]. Therefore, we speculated the beneficial impact of angiotensin-converting enzyme inhibitors (ACEIs) on arterial stiffness that extends beyond BP reduction [15]. Many studies illustrated the potential impact of ACEIs on arterial stiffness in various populations, and reported a protective role of ACEIs against arterial stiffness in hypertensive patients [16, 17]. Further, hypertension is characterized by thickened arterial wall, reduced blood vessel elasticity, and lumen dilatation prior to the rise in BP, which involves as vascular remodeling and is also a major cause of hypertensive complications. The structure and function of blood vessels should be restored in hypertensive patients receiving ACEIs, reducing the occurrence of cardiovascular and cerebrovascular events [18–21].

Arterial stiffness is predominantly reflected by the traveling speed of this pulse wave, and is termed as the pulse wave velocity (PWV) [22, 23]. According to the Europe Hypertension Management Guidelines that was published by the Society of Hypertension and the European Society of Cardiology (ESC/ESH) for the first time, PWV is included as one of the limb indicators for assessing subclinical target organ damage in hypertension [24]. Studies have emphasized the importance of applying PWV for representing arterial stiffness [8, 25–27]. Mallareddy et al. evaluated adult hypertensive patients without complex arterial structural or hemodynamic changes administered an ACEI for >4 weeks, and confirmed that ACEIs have a role in reducing PWV levels [28]. Meanwhile, Kithas and Supiano evaluated patients with essential hypertension otherwise in good general health (no underweight or morbid obesity) after 6 months of treatment, and confirmed the role of hydrochlorothiazide and spironolactone in lowering PWV levels [29]. However, there is a lack of latest meta-analysis results regarding the comparison of the effects of ACEIs versus placebo or other hypertensive agents on PWV (brachial-ankle PWV [ba-PWV] and carotid-femoral PWV [cf-PWV]) levels. Therefore, this meta-analysis was performed to assess the effectiveness of ACEIs on arterial stiffness.

## 2. Methods

### 2.1. Data Sources, Search Strategy, and Selection Criteria

This review was conducted and reported according to the guidelines of Preferred Reporting Items for Systematic Reviews and Meta-Analysis Statement issued in 2009 (Checklist S1) [30]. RCTs published in English and those that investigated the effectiveness of ACEIs on arterial stiffness were systematically searched from PubMed, Embase, and the Cochrane library till September 2018. There were no restrictions placed on publication status (published, in press, or in progress). The main search terms used were as follows: (“ramipril” or “cilazapril” or “quinapril” or “perindopril” or “lisinopril” or “captopril” or “Temocapril” or “quinapril” or “angiotensin-converting enzyme inhibitors” or “ACEI”) AND (“pulse wave velocity” or “PWV”) and are defined as “humans,” and “randomized controlled trials.” Furthermore, the references of eligible studies were manually searched for any eligible articles.

The literature search and study selection were undertaken by 2 authors independently by using a standardized approach. Any inconsistencies were settled by a corresponding author. Studies were considered eligible for inclusion if the following criteria were met: (1) studies with RCT design; (2) adult patients regardless of disease status; (3) the intervention was ACEIs and the control group was placebo or other hypertensive agents; and (4) the study should report PWV (ba-PWV and cf-PWV). The exclusion criteria were as follows: animal experiments and duplicated studies; studies designed as cross-over designs; and PWV was not categorized as ba- or cf-PWV.

### 2.2. Data Collection and Quality Assessment

All reliable and interested data of baseline characteristics and primary endpoints in the studies are extracted by two reviewers, respectively. In addition, if more than two arms compared the efficacy of ACEIs vs. other medications, all the relevant data associated with other medications should be pooled and interested endpoints were extracted and used to perform the pooled analysis. The data collected included the following information: first author's name, publication year, country, sample size, mean age, percentage male, baseline systolic blood pressure/diastolic blood pressure (SBP/DBP), intervention, control, follow-up duration, baseline PWV, and the PWV and SBP/DBP levels after interventions. The Jadad score was used to evaluate methodological quality of RCTs in this meta-analysis [31]. Briefly, the overall scale of a clinical trial was described from 0 to 5 if using the Jadad scale. In this meta-analysis, individual trials with scores 4 or 5 are regarded as high-quality studies.

### 2.3. Statistical Analysis

Continuous variables were presented as mean and standard deviation, the summary of weighted mean differences (WMDs) and 95% confidence intervals (CIs) were employed to calculate the effect of ACEIs on arterial stiffness, and SBP/DBP was evaluated by using random-effects model [32, 33]. The heterogeneity size was determined with *I*^2^ and *Q* statistics, and *P* value of less than 0.10 was considered as significant heterogeneity [34, 35]. Sensitivity analysis was conducted for investigating the outcomes to evaluate the impact of single study on overall analysis [36]. Subgroup analyses were conducted for ba-PWV, cf-PWV, SBP, and DBP based on country, mean age, percentage male, follow-up duration, baseline PWV, and study quality after excluding the study that included patients with normal blood pressure (mean SBP<140 mm Hg). Furthermore, interaction tests were performed to evaluate the differences between subgroups [37]. Funnel plots were drawn, and were used to perform Egger and Begg tests for the included articles for determining the presence of any publication bias [38, 39]. The test level for pooled results, sensitivity, subgroup, and publication bias was 0.05. Statistical analyses were performed by using STATA software (version 10.0; Stata Corporation, College Station, TX, USA).

## 3. Results

### 3.1. Literature Selection

**Figure 1:**
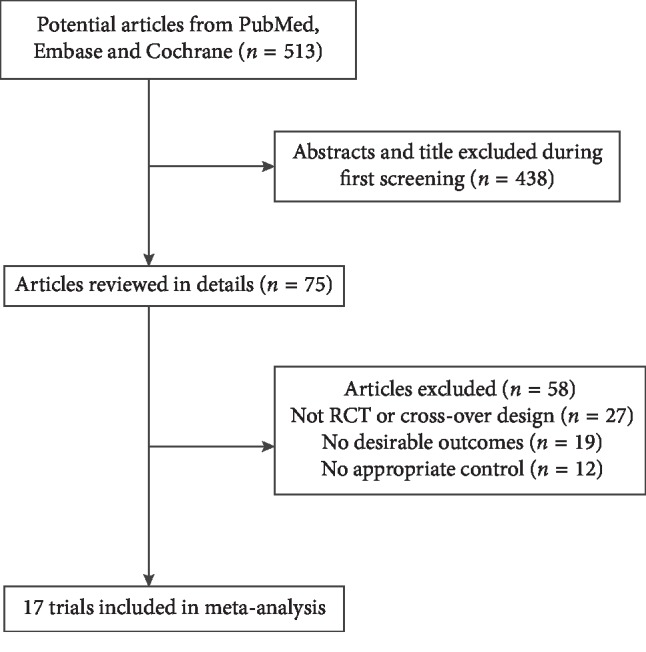
Flow chart of the study-selection process. Of the 513 hits retrieved in the initial database search, 438 were excluded by reviewing abstracts and titles, leaving 75 trials for assessment. After reviewing the full texts, 58 reports were excluded, and a total of 17 articles were included in this meta-analysis.

The search results of RCTs by the two reviewers were highly consistent. After utilizing the search strategy, a total of 513 published articles were retrieved. After screening the titles and abstracts according to the inclusion and exclusion criteria, 438 articles were discarded due to irrelevance of topic, animal studies, drug treatment, or improper evaluation of effectiveness and outcomes. Reading the full texts of the remaining 75 studies yielded 17 studies for inclusion, and 58 studies were excluded due to non-RCT or cross-over designs, no desired outcomes (ba-PWV and cf-PWV), or no appropriate control. Finally, 17 studies were selected for final meta-analysis [40–56]. The results of study-selection process are presented in Figure 1.

### 3.2. Characteristics of Included Studies

The general characteristics of each included study are listed in Table 1. There were 1,458 patients in the included RCTs, and their mean age ranged from 32.8 to 69.0 years. The included studies were conducted in Argentina, Japan, Poland, Malaysia, UK, Italy, Greece, and Spain. Among the included studies, 4 studies reported ba-PWV and 14 studies reported cf-PWV. Fifteen studies included hypertensive patients, and the remaining 2 studies included individuals with normal BP. The duration of intervention ranged from 8–48 weeks. Study quality was evaluated by using the Jadad scale, and 9 studies had a score of 4, and the remaining had a score of 3.

### 3.3. ba-PWV

**Figure 2:**
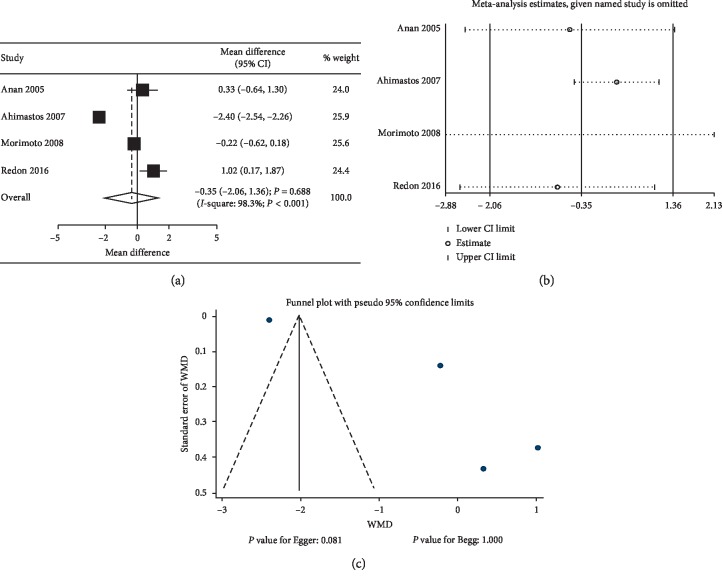
Effects of ACEIs on ba-PWV. (a) Summary of mean differences; (b) sensitivity analysis; (c) funnel plot for publication bias assessment, by Egger's and Begg's methods. CI, confidence interval; WMD, weighted mean difference; ACEIs, angiotensin-converting enzyme inhibitors; ba-PWV, brachial-ankle pulse wave velocity.

Data regarding the effect of ACEIs on ba-PWV were available from 4 studies. The summary results indicated no significant differences between ACEIs and control regarding the change of ba-PWV levels (WMD: −0.35; 95% CI: −2.06 to 1.36; *P*=0.688; Figure 2(a)), and significant heterogeneity was observed (*P* < 0.001). Furthermore, no significant differences were observed in hypertensive patients (WMD: 0.31; 95% CI:−0.48 to 1.10; *P*=0.443; substantial heterogeneity), and ACEIs were associated with lower ba-PWV levels in normal subjects (WMD: −2.40; 95% CI: −2.54 to −2.26; *P* < 0.001). Hence, a sensitivity analysis was conducted, while the conclusion was unaffected by sequential exclusion of any specific study (Figure 2(b)). Although country, mean age, percentage male, control, baseline PWV, and study quality might affect the treatment effects of ACEIs on ba-PWV levels (*P* < 0.05), subgroup analyses indicated that the ACEIs were associated with increased levels of ba-PWV if the study was conducted in Western countries (WMD: 1.02; 95% CI: 0.17 to 1.87; *P*=0.019), mean age of <60.0 years (WMD: 0.71; 95% CI: 0.04 to 1.39; *P*=0.037), percentage male ≥60.0% (WMD: 1.02; 95% CI: 0.17 to 1.87; *P*=0.019), compared with ARB (WMD: 0.71; 95% CI: 0.04 to 1.39; *P*=0.037), baseline PWV of <10.0 (WMD: 1.02; 95% CI: 0.17 to 1.87; *P*=0.019), and high-quality studies (WMD: 1.02; 95% CI: 0.17 to 1.87; *P*=0.019) (Table 2). The publication bias for ba-PWV was shown in Figure 2(c), and the Egger (*P*=0.081) or Begg test (*P*=1.000) results showed no significant publication biases for ba-PWV.

### 3.4. cf-PWV

**Figure 3:**
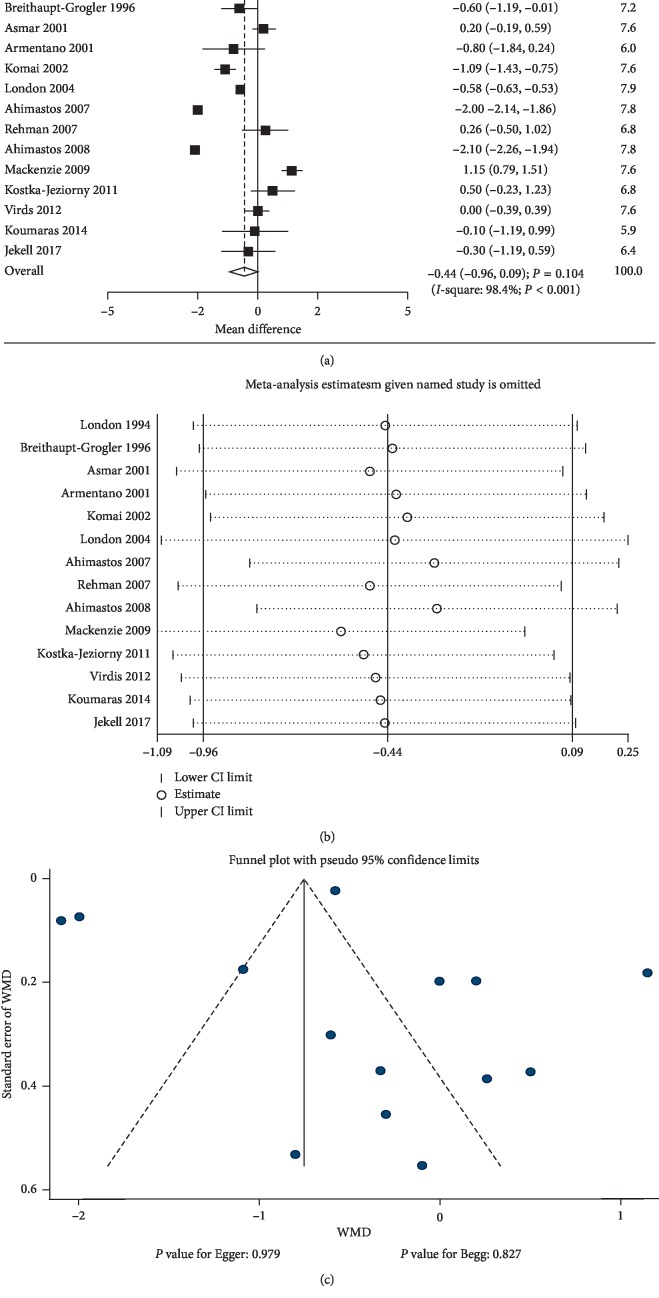
The results of the effect of ACEIs on cf-PWV. (a) Summary of mean differences; (b) sensitivity analysis; (c) funnel plot for publication bias assessment, by Egger's and Begg's methods. CI, confidence interval; WMD, weighted mean difference; ACEIs, angiotensin converting enzyme inhibitors; cf-PWV, carotid-femoral pulse wave velocity.

Data regarding the effects of ACEIs on cf-PWV were available from 14 studies. There was no significant difference between ACEIs and control for cf-PWV level (WMD: −0.44; 95% CI: −0.96 to 0.09; *P*=0.104; Figure 3(a)). Heterogeneity was observed in the magnitude of the effect across the trials (*P* < 0.001). No significant difference was observed in hypertensive patients (WMD: −0.13; 95% CI: −0.54 to 0.27; *P*=0.528; substantial heterogeneity), and ACEI was associated with lower cf-PWV levels in normal individuals (WMD: −2.05; 95% CI: −2.15 to −1.94; *P* < 0.001; without evidence of heterogeneity). The sensitivity analysis results indicated change in the pooled results after excluding the study conducted by Mackenzie et al., [51] which specifically included elderly patients (Figure 3(b)). Subgroup analysis indicated that the ACEIs were associated with lower levels of cf-PWV when the percentage male was ≥60.0% (WMD: −0.40; 95% CI: −0.75 to −0.04; *P*=0.031) and studies with high-quality scores (WMD: −0.41; 95% CI: −0.79 to −0.03; *P*=0.032) (Table 3). *P* value for interaction tests indicated that country, mean age, percentage male, control, follow-up duration, baseline PWV, and study quality could bias the therapeutic effects between ACEI and control groups (*P* < 0.05). There was no significant publication bias for cf-PWV (*P* value for Egger: 0.979; *P* value for Begg: 0.827; Figure 3(c)).

### 3.5. SBP and DBP

**Figure 4:**
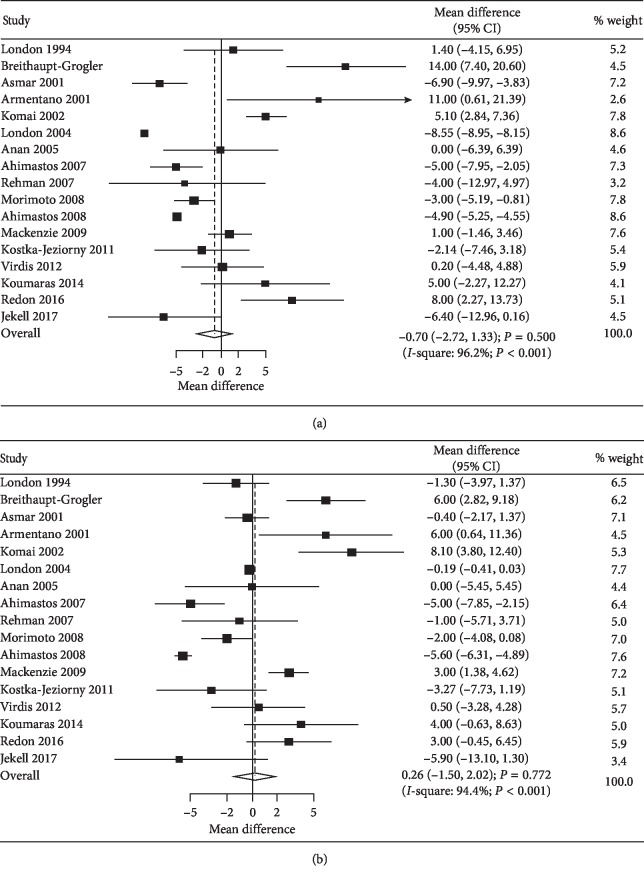
Effects of ACEIs on SBP (a) and DBP (b). Weighted mean differences are provided as well as 95% confidence intervals (CIs). SBP, systolic blood pressure; DBP, diastolic blood pressure.

All the included studies reported SBP and SBP at baseline and after interventions. The summary WMD indicated that patients who received ACEI showed no significant difference in SBP compared with the control group (WMD: −0.70; 95% CI: −2.72 to 1.33; *P*=0.500; Figure 4(a)). Moreover, significant heterogeneity was observed among the included trials. Furthermore, no significant difference was observed between ACEI and control groups regarding the change of DBP (WMD: 0.26; 95% CI: −1.50 to 2.02; *P*=0.772; Figure 4(b)), and substantial heterogeneity was detected. Subgroup analyses indicated that ACEI was associated with greater reduction in SBP when the follow-up duration was >24.0 weeks (WMD: −4.43; 95% CI: −8.61 to −0.25; *P*=0.038), whereas ACEI was associated with higher SBP when the baseline PWV was <10 (WMD: 3.67; 95% CI: 0.11 to 7.23; *P*=0.043; Table 4). Also, ACEI was associated with greater reduction in DBP when compared with CCB (WMD: −1.74; 95% CI: −3.38 to −0.10; *P*=0.038), whereas the reduction in DBP in patients who received ACEIs was smaller when compared with BRB (WMD: 2.57; 95% CI: 0.39 to 4.74; *P*=0.021), and baseline PWV was <10 (WMD: 3.30; 95% CI: 1.10 to 5.50; *P*=0.003; Table 5).

## 4. Discussion

In this meta-analysis, the effects of ACEIs on arterial stiffness based on RCTs and changes of PWV (ba-PWV and cf-PWV) were analyzed. Combined data of ba-PWV and cf-PWV levels served as a reflection of the degree of atherosclerosis in the upper-limb and lower-limb indicators. The results of our study showed no significant differences between ACEIs and control in improving the vascular stiffness, including ba-PWV and cf-PWV. Subgroup analyses indicated that the therapeutic effects of ACEIs on ba-PWV could affect by country, mean age, percentage male, control, baseline PWV, and study quality, while cf-PWV levels might be affected by country, mean age, percentage male, control, follow-up duration, baseline PWV, and study quality. Moreover, BP reduction in patients who received ACEIs and controls showed no significant difference.

Numerous meta-analyses have investigated the impact of ACEIs on arterial stiffness [17, 28, 57]. Mallareddy et al. [28] performed a meta-analysis to investigate the impact of ACEIs on PWV or augmentation index. The results revealed that reduction in PWV was −1.15 m/s for cf-PWV and −1.9 m/s for ba-PWV for patients who received ACEIs. Furthermore, they pointed out that ACEIs had modest beneficial influence on arterial stiffness, which was partly independent of the changes in BP. [28] Shahin et al. examined the effects of ACEIs on arterial stiffness and wave reflections in patients with hypertension. The results revealed that ACEIs vs. placebo showed significantly reduced levels of PWV, while comparison with other antihypertensive drugs showed no significant differences in PWV levels. Moreover, ACEIs reduced PWV levels in patients with different pathological conditions [57]. Xie et al. conducted a meta-analysis based on 8 RCTs and concluded no significant differences between ACEIs and atenolol on the levels of ba-PWV and cf-PWV. Also, ACEIs were inferior over atenolol in peripheral DBP and heart rate, while the levels of peripheral SBP between ACEIs and atenolol showed no significant difference [17]. However, there are several limitations in the previous meta-analyses studies that should be mentioned: (1) various designs of included studies might affect the therapeutic effects of ACEIs on arterial stiffness; (2) the studies included designs of cross-over trial, and had different washout periods, which in turn could bias the effectiveness of ACEIs; (3) the summary results of PWV were combined, and the type of ba-PWV and cf-PWV was analyzed, respectively; and (4) the therapeutic effects of ACEIs on arterial stiffness in patients with specific characteristics were not illustrated. Therefore, this meta-analysis was conducted based on RCTs to minimize the abovementioned limitations to ensure the confidence of our results.

From the funnel plots of the above two analyses, the study using PWV as detection index was less likely to be biased. Therefore, the conclusions of this study will be able to reflect changes in vascular function. Furthermore, the results of this study showed no significant differences between ACEIs and control for the indexes of arterial stiffness. The possible reason for this could be due to the use of various antihypertensive drugs, and the net therapeutic effects of ACEIs among the included trials varied. Moreover, the summary results and 95% CIs of ba-PWV and cf-PWV were affected by the WMD of individual trial. In addition, the treatment effects of ACEI on arterial stiffness could affect the effect size of BP changes. This study showed no significant differences between ACEIs and control regarding the changes of SBP and DBP, suggesting that the therapeutic effects of ACEIs on arterial stiffness were not biased by the changes of SBP and DBP.

The significant therapeutic effects of ACEIs were observed for ba-PWV when compared with ARB or placebo (Table 2). These results indicated that ARB provided superior effects on ba-PWV than ACEIs. Also, ACEIs showed greater reduction in ba-PWV when compared with placebo (Table 2). In addition, the therapeutic effects of ACEIs on cf-PWV levels were affected by country, mean age, percentage male, control, follow-up duration, baseline PWV, and study quality (Table 3). Firstly, the background therapies and lifestyles were correlated with country and mean age, and were associated with the therapeutic effects of ACEI; secondly, the differences in risk stratification between men and women could affect the levels of arterial stiffness; thirdly, the type of control drugs was correlated with the net therapeutic effects between the intervention and control groups; fourthly, the baseline PWV level was associated with the severity of arterial stiffness; and the study quality was correlated with the evidence level and reliability of summary results. However, the results of subgroup analyses were considered unreliable due to the stability of pooled results and substantial heterogeneity within the subgroups.

Although the study was professionally conducted, there were still a few shortcomings that need to be noted. (1) The purpose of this study was to analyze the curative effects of ACEIs on hypertensive patients by meta-analysis. Some studies did not mention the basis for estimating the sample size or did not fully implement the blinding method, and the study sample size was not large enough, impacting on the test results to a certain extent. (2) There were specific differences in the clinical design, type of drug, dose, follow-up time, etc. The usage of ACEIs varied between the included studies, as well as administration of drugs and the duration of intervention, contributing to the heterogeneity of subgroup analysis of the reported endpoints. In addition, other antihypertensive drugs were used as controls. (3) The relatively small number of included studies when combined with large sample clinical trials and small sample size test may lead to bias.

## 5. Conclusions

Taken together, although there are some limitations, the strengths and inferences of the overall conclusion are overwhelming. Meta-analysis of RCTs suggested that the role of ACEIs in improving arterial stiffness in hypertensive patients were observed in several subsets. These conclusions may not be applicable to the overall populations, and more evidences are needed to compare the prognosis of patients taking ACEIs with other drugs. Future large-scale studies to verify the results of subgroup analyses in this meta-analysis should be conducted.

## Figures and Tables

**Table 1:** Baseline characteristics of included studies.

Studies	Country	Number of patients	Mean age (years)	Percentage male (%)	Baseline SBP/DBP (mmHg)	Intervention	Control	Follow-up duration	PWV type	Baseline PWV (m/s)	Jadad scale
London et al. [40]	France	24	53.5	58.3	176.9/98.5	Perindopril	Nitrendipine	48 weeks	cf-PWV	13.0	3
Breithaupt-Grogler et al. [41]	Germany	17	45.0–67.0	NA	149.9/98.3	Cilazapril	Hydrochlorothiazide	12 weeks	cf-PWV	9.2	3
Asmar et al. [42]	Australia	406	18.0–84.0	NA	162.2/98.7	Indapamide and perindopril	Atenolol	48 weeks	cf-PWV	12.3	4
Armentano et al. [43]	Argentina	34	55.0	64.7	157.0/95.0	Ramipril	Atenolol	12 weeks	cf-PWV	11.7	4
Komai et al. [44]	Japan	25	62.1	64.0	152.0/81.9	Cilazapril	Atenolol	24 weeks	cf-PWV	9.5	4
London et al. [45]	Australia	469	54.9	67.4	162.3/98.6	Indapamide and perindopril	Atenolol	48 weeks	cf-PWV	12.7	4
Anan et al. [46]	Japan	21	59.0	47.6	156.5/97.0	Perindopril	Valsartan	40 weeks	ba-PWV	18.3	3
Ahimastos et al. [47]	Australia	17	32.8	76.5	120.4/72.2	Perindopril	Placebo	24 weeks	cf-PWV and ba-PWV	7.4	4
Rehman et al. [48]	Malaysia	39	52.8	NA	151.5/93.0	Perindopril	Losartan	24 weeks	cf-PWV	11.5	3
Morimoto et al. [49]	Japan	32	64.0	46.9	145.5/82.0	Perindopril	Amlodipine	24 weeks	ba-PWV	16.4	3
Ahimastos et al. [50]	Australia	40	66.0	95.0	139.5/87.0	Ramipril	Placebo	24 weeks	cf-PWV	9.7	4
Mackenzie et al. [51]	United Kingdom	32	69.0	50.0	159.4/84.1	Perindopril	Atenolol	10 weeks	cf-PWV	9.3	3
Kostka-Jeziorny et al. [52]	Poland	66	46.2	60.6	158.2/96.8	Perindopril	Hydrochlorothiazide	8 weeks	cf-PWV	11.2	3
Virdis et al. [53]	Italy	50	45.0	70.0	148.4/92.8	Ramipril	Aliskiren	12 weeks	cf-PWV	7.6	4
Koumaras et al. [54]	Greece	37	48.0	78.4	148.4/99.4	Quinapril	Atenolol	10 weeks	cf-PWV	8.8	3
Redon et al. [55]	Spain	88	58.4	60.2	157.5/96.5	Perindopril	Olmesartan	24 weeks	ba-PWV	9.2	4
Jekell et al. [56]	Sweden	61	53.6	65.6	152.5/92.9	Ramipril	Doxazosin	12 weeks	cf-PWV	8.7	4

PWV, pulse wave velocity; ba-PWV, brachial-ankle pulse wave velocity; cf-PWV, carotid-femoral pulse wave velocity; SBP, systolic blood pressure; DBP, diastolic blood pressure; NA, not available.

**Table 2:** Subgroup analyses for ba-PWV.

Group	WMD and 95% CI	*P* value	Heterogeneity (%)	*P* value for heterogeneity	*P* value for interaction test
Country					
Eastern	−0.13 (−0.53 to 0.27)	0.527	6.1	0.302	0.014
Western	1.02 (0.17 to 1.87)	0.019	—	—	

Mean age (years)					
≥60.0	−0.22 (−0.62 to 0.18)	0.275	—	—	0.014
<60.0	0.71 (0.04 to 1.39)	0.037	9.3	0.294	

Percentage male (%)					
≥60.0	1.02 (0.17 to 1.87)	0.019	—	—	0.014
<60.0	−0.13 (−0.53 to 0.27)	0.527	6.1	0.302	

Control					
ARB	0.71 (0.04 to 1.39)	0.037	9.3	0.294	<0.001
CCB	−0.22 (−0.62 to 0.18)	0.275	—	—	

Follow-up duration					
>24	0.33 (−0.64 to 1.30)	0.504	—	—	0.530
≤24	0.34 (−0.87 to 1.55)	0.581	85.1	0.010	

Baseline PWV					
≥10.0	−0.13 (−0.53 to 0.27)	0.527	6.1	0.302	0.014
<10.0	1.02 (0.17 to 1.87)	0.019	—	—	

Study quality					
High	1.02 (0.17 to 1.87)	0.019	—	—	0.014
Low	−0.13 (−0.53 to 0.27)	0.527	6.1	0.302	

PWV, pulse wave velocity; ba-PWV, brachial-ankle pulse wave velocity; CCB, calcium channel blocker; CI, confidence interval; ARB, angiotensin receptor blocker or inhibitor; DBP, diastolic blood pressure; WMD, weighted mean difference.

**Table 3:** Subgroup analyses for cf-PWV.

Group	WMD and 95% CI	*P* value	Heterogeneity (%)	*P* value for heterogeneity	*P* value for interaction test
Country					
Eastern	−0.46 (−1.78 to 0.86)	0.496	90.1	0.001	0.042
Western	−0.06 (−0.54 to 0.42)	0.814	92.5	<0.001	

Mean age (years)					
≥60.0	0.03 (−2.17 to 2.22)	0.979	98.7	<0.001	<0.001
<60.0	−0.18 (−0.53 to 0.17)	0.312	69.5	0.002	

Percentage male (%)					
≥60.0	−0.40 (−0.75 to −0.04)	0.031	77.5	<0.001	<0.001
<60.0	0.45 (−1.00 to 1.89)	0.547	92.2	<0.001	

Control					
ARB	0.01 (−0.31 to 0.33)	0.961	0.0	0.642	0.003
CCB	−0.33 (−1.06 to 0.40)	0.373	—	—	
Diuretic	−0.07 (−1.15 to 1.01)	0.896	81.0	0.022	
BRB	−0.18 (−0.85 to 0.48)	0.588	95.6	<0.001	

Follow-up duration					
>24	−0.25 (−0.83 to 0.33)	0.399	87.4	<0.001	<0.001
≤24	−0.10 (−0.72 to 0.53)	0.764	90.8	<0.001	

Baseline PWV					
≥10.0	−0.12 (−0.59 to 0.35)	0.615	82.7	<0.001	<0.001
<10.0	−0.15 (−0.97 to 0.67)	0.719	93.9	<0.001	

Study quality					
High	−0.41 (−0.79 to −0.03)	0.032	84.9	<0.001	<0.001
Low	0.17 (−0.50 to 0.85)	0.615	84.7	<0.001	

PWV, pulse wave velocity; cf-PWV, carotid-femoral pulse wave velocity; CCB, calcium channel blocker; CI, confidence interval; ARB, angiotensin receptor blocker or inhibitor; DBP, diastolic blood pressure; WMD, weighted mean difference.

**Table 4:** Subgroup analyses for SBP.

Group	WMD and 95% CI	*P* value	Heterogeneity (%)	*P* value for heterogeneity	*P* value for interaction test
Country					
Eastern	−0.05 (−5.50 to 5.40)	0.985	88.7	<0.001	<0.001
Western	0.98 (−3.47 to 5.42)	0.667	94.6	<0.001	

Mean age (years)					
≥60.0	1.03 (−3.71 to 5.76)	0.671	92.1	<0.001	<0.001
<60.0	0.09 (−4.72 to 4.89)	0.972	90.6	<0.001	

Percentage male (%)					
≥60.0	1.19 (−5.17 to 7.56)	0.713	96.6	<0.001	<0.001
<60.0	−0.55 (−3.16 to 2.06)	0.678	54.2	0.088	

Control					
ARB	−0.09 (−4.90 to 4.73)	0.972	66.2	0.019	<0.001
CCB	−1.57 (−5.61 to 2.47)	0.446	52.1	0.149	
Diuretic	5.81 (−10.01 to 21.62)	0.472	92.8	<0.001	
BRB	0.41 (−5.98 to 6.80)	0.900	97.6	<0.001	

Follow-up duration					
>24	−4.43 (−8.61 to −0.25)	0.038	85.0	<0.001	<0.001
≤24	2.25 (−0.90 to 5.40)	0.162	82.7	<0.001	

Baseline PWV					
≥10.0	−2.75 (−6.19 to 0.70)	0.118	88.	<0.001	<0.001
<10.0	3.67 (0.11 to 7.23)	0.043	79.2	<0.001	

Study quality					
High	−0.14 (−6.27 to 6.00)	0.965	96.9	<0.001	<0.001
Low	1.24 (−2.06 to 4.54)	0.461	75.4	<0.001	

ARB, angiotensin receptor blocker or inhibitor; CCB, calcium channel blocker; CI, confidence interval; PWV, pulse wave velocity; SBP, systolic blood pressure; WMD, weighted mean difference.

**Table 5:** Subgroup analyses for DBP.

Group	WMD and 95% CI	*P* value	Heterogeneity (%)	*P* value for heterogeneity	*P* value for interaction test
Country					
Eastern	1.18 (−3.54 to 5.90)	0.624	82.7	0.001	0.910
Western	1.19 (−0.33 to 2.71)	0.125	78.0	<0.001	

Mean age (years)					
≥60.0	2.70 (−1.98 to 7.39)	0.258	91.4	<0.001	0.004
<60.0	0.24 (−1.21 to 1.69)	0.744	46.1	0.054	

Percentage male (%)					
≥60.0	1.69 (−0.80 to 4.19)	0.184	76.7	<0.001	0.162
<60.0	−0.00 (−2.96 to 2.95)	0.998	81.8	0.001	

Control					
ARB	0.25 (−2.15 to 2.65)	0.839	26.8	0.243	0.026
CCB	−1.74 (−3.38 to -0.10)	0.038	0.0	0.685	
Diuretic	1.50 (−7.58 to 10.58)	0.746	70.9	0.001	
BRB	2.57 (0.39 to 4.74)	0.021	86.4	<0.001	

Follow-up duration					
>24	−0.20 (−0.42 to 0.02)	0.074	0.0	0.870	<0.001
≤24	1.89 (-0.34 to 4.12)	0.096	77.7	<0.001	

Baseline PWV					
≥10.0	−0.58 (−1.55 to 0.40)	0.249	34.2	0.155	<0.001
<10.0	3.30 (1.10 to 5.50)	0.003	62.9	0.013	

Study quality					
High	1.51 (−0.53 to 3.55)	0.146	76.2	<0.001	0.020
Low	0.75 (−1.60 to 3.11)	0.530	78.2	<0.001	

ARB, angiotensin receptor blocker or inhibitor; CCB, calcium channel blocker; CI, confidence interval; PWV, pulse wave velocity; DBP, diastolic blood pressure; WMD, weighted mean difference.
